# Supplementary material for: Are carbon emissions trading and green financial instruments synergistic? -Comprehensive quantitative research based on content analysis
Source: PLoS One. 2024 Mar 7;19(3):e0298601. doi: 10.1371/journal.pone.0298601 (PMC10919601; doi:10.1371/journal.pone.0298601)
Supplement: S1 Data — (ZIP) [file pone.0298601.s001.zip › Data/地级市Z_F工作报告.zip!/║┌┴·╜¡/║┌║╙╩╨/└┤╫╘╣1⁄2╓┌║┼í╛┬φ┐╦╩2╛▌═°í┐_2016.docx]

各位代表：

现在，我代表黑河市人民政府，向大会作工作报告，请予审议，并请列席会议的同志提出宝贵意见。

一、2015年和“十二五”工作回顾

2015年是“十二五”收官之年，我们在省委、省政府和市委正确领导下，适应经济新常态，坚定发展信心，主动担当作为，全力打好“三大攻坚战”，实施“六大提升工程”，经济社会平稳健康发展。预计全市生产总值450亿元，同比增长7.5%。全社会固定资产投资256亿元，增长5%。社会消费品零售总额104亿元，增长9%。公共财政收入28.9亿元，增长9.8%。城镇常住居民人均可支配收入23201元，农村常住居民人均可支配收入12598元，分别增长10%和10.5%。万元地区生产总值综合能耗下降2%以上。

——“三大攻坚战”成效显著

坚持打好产业项目攻坚战，内生动力明显增强。89个重点产业项目开复工87个，完成投资91亿元。19个省重点产业项目开复工17个，完成投资35亿元。亿元以上项目投产10个，占新投产项目三分之一。新开工项目56个，完成投资占重点项目投资60%。借助“外脑”谋划项目，与中国农科院、北京交通大学等科研院所和高校对接，新谋划一批专业化、高水准大项目。加快产业园区建设，形成了以黑河进出口加工产业园、黑河(北安、孙吴)健康产业园为主体，各类园区优势互补的发展格局。北安华海光伏从签约到开工只用48天，象屿金谷当年立项、当年建设、当年投产、当年缴税，创造了项目建设的“北安速度”。北安经济开发区、五大连池风景区生态矿泉产业示范基地晋升为省级经济开发区。广泛开展招商活动，把央企和大型民企作为主攻方向，赴北京、上海、南京、深圳、香港等地推介，落实项目263个，到位资金201.6亿元。创新融资方式，在全省地级市中率先设立2亿元产业引导股权投资基金，与北京银建集团合作组建了首支农林产业子基金，与深圳前海两型基金就组建基础设施产业子基金达成合作共识。北亿农业科技在上海股交中心E板挂牌上市，成为我省首家E板上市企业。五大连池名镇上市进入终审，金禾经贸上报新三板监管部门审批，中兴牧业进入资产审计评估阶段。鑫盛担保公司与深圳“鹏金所”合作，开展P2B互联网金融服务模式，爱辉区义鑫融资担保公司投入运营。农村物权融资平台组建完成。嫩江开展了农村合作金融公司试点，逊克农村信用信息查询平台试点完成。鼓励金融机构扩大信贷规模，投放企业贷款增长10%。煤矿整顿关闭方案在全省第一个获得批复。启动“千户科技型企业三年行动计划”，培育科技型企业20户。新增规模以上工业企业3户。县域经济实力不断增强，北安荣获“全省财源建设十强县”，嫩江荣获“全省经济发展十强县”，孙吴、逊克荣获“全省县域经济发展质量效益优秀县”荣誉称号。

坚持打好重大基础设施建设攻坚战，发展基础更加坚实。黑龙江大桥、跨江空中索道前期取得突破，在两国元首共同见证下，中俄正式签署修订建桥协定议定书和索道协定。五大连池机场在全省在建机场中率先开工。水利工程建设经受住严峻考验，11类96项工程完成投资20.5亿元，是上年的2.6倍，创历史之最。黑河四岛保护开发工程完成投资5.7亿元，占总投资96.5%，三年工期一年完成。三江治理工程在全省率先开工，黑龙江干流治理完成198公里，嫩江干流治理完成17.8公里，得到水利部和省委、省政府高度认可。黑河市区及孙吴、逊克、嫩江城区防洪工程进展顺利，中小河流治理、水库除险加固工程开工，水电站扩容改造、国土防护工程完工。启动旱田节水灌溉和灌区工程，新增灌溉面积26万亩。北黑铁路改造升级和北五铁路建设项目前期加快推进。新建农村公路426公里，全市566个行政村全部通车。投资7000余万元修复国省干线公路255公里，2013年特大洪水冲毁的道路设施全面修复。投资1亿元全面完成公路平交道口改造任务。引导社会资本建设储粮和烘干设施，新增仓容15万吨，新建烘干塔36座，粮食储备能力进一步提高。

坚持打好城市建设攻坚战，城市品位不断提升。“三城同创”取得突破，国家卫生城、国家园林城创建成功，国家环保模范城通过省级初检，全国文明城获得提名资格。强化城市基础设施配套，口岸货运浮箱固冰通道道路连接线、四中北侧道路西段项目启动建设，江天路、桦林路、民益街完工。实施街路、庭院、小区绿化工程，对世纪广场、大黑河岛桥头进行多彩立体绿化，栽植乔灌木4.2万株，新增绿地14.4公顷，绿地率达到35.1%。加快“三供三治”项目建设，改造老旧供热管网16.2公里。净水厂工艺改造工程启动，水源地污染防治、海澜热电调峰热源项目完工，生活垃圾处理厂填埋区膜覆盖和防渗膜保护工程开工，污泥处理厂投入使用。开展地下管线普查。加大保障性安居工程建设力度，全市改造棚户区3372套、农村泥草(危)房4131户，市区最大的南部棚户区改造工程开工。市区维修改造老旧住宅楼90栋，提升改造旧小区30个。加强城市精细化管理，开展违法建设、私搭乱建专项整治，归集整理71个小区通讯管线。各县(市)城乡建设步伐加快，北安铁路下交桥完工，嫩江污水处理厂二期投入使用，五大连池滨水新城道路工程开工，孙吴道路硬化工程竣工，逊克楼房立面改造取得成效。打造美丽乡村，推进“三带百村工程”升级，56个重点村环境明显改善。

——“六大提升工程”有力推进

深入实施高纬寒地特色农业提升工程，现代农业加快发展。坚持用现代发展理念引领农业，积极推进“互联网+农业”，淘宝特色中国·黑河馆销售额突破千万元，阿里巴巴集团农村淘宝黑龙江省北部运营总部落户北安。开发绿色特色产品，发展有机大豆、青贮玉米、水稻和汉麻、饲草等优质高效作物，绿色有机农产品认证品种达到81个。加快现代牧场建设，省部级标准化示范场达到13家。中兴牧业新建牧场4个，奶牛存栏2.5万头，乳制品加工项目完成选址。发展生态特色渔业，水产品养殖面积51.3万亩。发展林业特色产业，实现产值40亿元，增长20%。北药面积达到58万亩，小浆果栽培达到10万亩。壮大农产品加工企业，引进广州恒大、香港华章、上海正大等企业集团，开发功能性水稻和绿色有机产品，新增年产值千万元以上企业5家。强化合作社经营管理，8家合作社晋升为省级规范社，现代农机合作社达到141家。在全省率先建成农机管理调度指挥中心，实现省市县三级互联互通。扎实推进土地确权登记工作，北安通北镇国家级试点、嫩江科洛镇省级试点全面完成。

深入实施对俄合作转型提升工程，贸易投资深度拓展。成功举办2015中俄双子城展览会。发展跨境电子商务，建设跨境电子商务产业园区，电商企业达到520家，年销售额4.6亿元，黑河中机公司被评为国家级电子商务示范企业，“互联网+对俄合作”走在全省前列。加快对俄重点项目和境内外园区建设，阿穆尔—黑河边境油品储运与炼化综合体项目列入阿州别列佐夫卡超前发展经济区，星河公司年产60万吨水泥粉磨站投产。中俄东线天然气管道工程开工，萨哈油田区块开发启动勘探。布市汽车展销及售后服务中心主体工程完工，利源达集团境外机电营销项目在俄代理商达到21家。黑河进出口加工产业园引进俄电33亿度，阿州石化建材加工园区、北丰农业产业园被商务部列入在建境外园区企业名单。推进对俄农林合作，境外开发土地115万亩，回运大豆20万吨，进境返销粮食居全省首位，逊克中俄农业科技合作园引进良种39个，阿州和兴物流建材加工产业园农产品仓储批发销售区投入使用。成功举办第八届中俄林业生态建设学术论坛。对俄交往向俄腹地延伸，与雅库茨克市结为友好城市，与斯塔夫罗波尔边疆区达成合作共识。互市贸易交易点投入使用，1.2万边民享受政策红利。加强口岸建设，新旅检大厅完工，优化服务流程，延长工作时间，通关环境进一步改善。

深入实施旅游形象提升工程，消费经济有效拉动。全市接待旅游者649.2万人次，增长16.4%。旅游收入52.7亿元，增长15.3%。黑河游客服务中心投入运营，成为中俄边境线上最大的游客服务平台。智慧旅游公共服务平台实现在线交易，中俄文信息声讯热线开通。强化宣传促销，通过国内28个一类城市机场、2670组高铁列车全面展示旅游形象。联合《星光大道》开展主题公益活动，邀请《走遍中国》拍摄的专题节目在央视播出。在新浪、搜狐、优酷等大型网站推广黑河微电影，浏览人次突破6000万。五大连池风景区进入中国智慧旅游景区百强，被评为国家生态旅游示范区和全国体育旅游精品景区。五大连池大沾河国家森林公园晋升国家4A级景区，青山公园被评为国家级火山城市湿地公园。乡村旅游成为新亮点，爱辉区新生村、逊克大平台村、五大连池风景区清泉村被评为中国乡村旅游模范村。爱辉区红河谷国际滑雪场、嫩江塔溪生态园、孙吴胜山要塞、逊克库尔滨漂流等景区景点设施进一步完善。开展了雪地民俗体验、浪漫赴俄冬之旅等活动，开通了黑河至韩国旅游线路。黑河获得第21届亚洲旅游业金旅奖首批十佳健康(养生)旅游目的地、首批最美生态旅游目的地殊荣。

深入实施文化产业提升工程，城市文化内涵逐步彰显。突出中俄边境地域特色，完成“一江两岸四岛文化旅游圈”项目策划。与阿州共同举办庆祝世界反法西斯战争胜利70周年系列纪念活动。成功举办第六届中俄文化大集，国际影响力不断提升，被俄联邦文化部评为远东及西伯利亚地区最有魅力的文化交流项目。大型中俄风情歌舞《江水霓裳》演出46场，观众1.5万人次。搭建文化产业平台，与全国工商联旅游业商会签订合作框架协议，整体开发大黑河岛贸易旅游文化项目。中国书画艺术院黑河分院基础设施完工，中俄宝玉石产业园、逊克玛瑙文化产业园启动前期。加快文化设施建设，北安全省首家新文化广场工程进展顺利，文化综合体主体工程完工，文化艺术基地被列为省级重点产业项目，完成了国家级公共文化服务体系示范项目建设。知青博物馆完成改陈提升，《与共和国同命运》全国巡展北京展成功举办，知青影视播放厅、知青油画馆和老知青接待中心投入使用。黑河博物馆整体改造完工，特拨馆藏文物完成鉴定评估。黑河学院中俄(苏)抗日战争历史陈列馆建成，孙吴侵华日军罪证陈列馆开馆，瑷珲海关历史陈列馆正在布展。北方影视城影响力不断提升，电视剧《大江作证》在省电视台播放，《黑河风云》完成电视制作。弘扬民俗文化，五大连池“圣水节”、爱辉区“古伦木沓节”、嫩江“墨尔根古道驿站”形成特色文化品牌。桦树皮制作及镶嵌画作品入选在台湾举办的黑龙江省民间民俗展。3人获省首届非物质文化遗产薪传奖，北安乌鱼绣项目获省级非物质文化遗产研究基地称号。

深入实施生态建设提升工程，生态屏障功能进一步增强。加大天然林保护政策争取力度，重点公益林生态效益补偿资金标准大幅提高。大力开展造林绿化，完成造林20.7万亩、森林抚育42.4万亩。深入实施“一退三还”行动，还林、还草、还湿11万亩。探索碳汇林业发展有效途径，提高社会认知度和参与度，碳汇造林成果进一步巩固。推进森林湿地公园建设，乌裕尔、坤河、圈河湿地公园申报国家级工作积极推进。开展跨境生态保护合作，干岔子自然保护区与阿穆尔自然保护区实现对接。提高森林资源管护能力，连续19个防期未发生重特大森林火灾。黑河林业信息中心和数据共享平台投入运行，森林生态站项目获得国家批复。推广保护性耕作，测土配方施肥推广率73.1%，完成秸秆还田700万亩、深松整地365万亩。强化节能减排工作，超额完成五年节能目标任务。加强污染物减排和大气污染防治，淘汰黄标车和老旧车辆5224辆，市区供热燃煤小锅炉全部拆除，环境质量居全省前列。推进矿山环境治理，投资5000余万元，完成宋集屯、西岗子煤矿环境治理试点项目，整治土地282公顷，整理出耕地120公顷，植树绿化76公顷。三道湾子金矿、一五一和富宏煤矿国家级“绿色矿山”试点启动实施。

深入实施生活品质提升工程，群众幸福指数显著提高。加大民生投入，全市民生支出占财政支出69.8%，比上年提高12.5个百分点。激发大众创业、万众创新活力，强化政府引导扶持，发挥企业主体作用，举办了首届青年创新创业大赛，为大学生创业搭建梦想平台，黑河小微企业科技创业园、黑河市大学生创业孵化基地、爱辉区创青国际中俄青年创业中心、合作区双子城创客空间等创业平台如雨后春笋蓬勃兴起，吸引了一批本土和外地青年投身创业热潮。深入实施“30周岁以下高校毕业生就业工程”，安置高校毕业生1518人。城镇新增就业2万人，登记失业率3.48%，低于省定目标1.02个百分点。促进义务教育均衡发展，爱辉区、孙吴县域内校际间达到初步均衡标准。全市农村和城镇学校全部实现宽带接入，教育信息化水平居全省前列。加快校园“三化”建设，城镇学校校园全部实现合理硬化。高考成绩实现“四连增”，全市文理二批次以上上线率达到60%，比上年提高10.3个百分点。2名考生分别考入清华大学、北京大学。高职升学考试录取率100%，特殊教育残疾儿童入学率86.1%。推进“名优校长名优教师培养工程”，选拔知名校长30名、知名教师100名。深入开展防火、防震和交通安全知识进课堂活动。深化医药卫生体制改革，基层医疗机构基本药物制度实现全覆盖。加大医疗人才培养力度，评选出黑河名医25名、名中医4名。打造动感城市，举办了市第二届全民健身运动会，承办了中俄冰雪越野拉力赛和中俄橡皮艇极限挑战赛，中俄横渡黑龙江活动上升为国家级赛事。竞技体育取得突破，获得省级以上赛事奖牌27枚，获得短道速滑世界杯比赛奖牌3枚。全民健身中心体育场投入使用，体育馆和体育公园开工建设，社区多功能运动场、网球场向市民开放。深入实施“双百”示范工程，城市社区达标率98%，农村社区启动率92%。提高社会保障水平，城镇居民医疗保险住院医疗费最高支付限额由6万元提高到8万元。建立城镇居民大病医疗保险制度，城镇职工(居民)医疗保险实现市级统筹。实施开发式扶贫，全市完成14个贫困村整村推进项目建设，减少贫困人口5230人。与中国地震应急搜救中心合作建设高寒试验场，填补了国内国际空白。立体化社会治安防控体系、公共安全治理体系不断完善，边境反恐工作进一步加强。安全生产形势稳定，信访形势总体平稳，边境管理得到加强。民族宗教、侨务、妇女儿童、人防、档案、地方史志等工作也都取得新成绩。

——政府系统“三严三实”专题教育扎实开展

巩固群众路线教育实践活动成果，践行“三严三实”要求，突出问题导向，加大整改力度，政府自身建设进一步强化。主动接受人大依法监督和政协民主监督，181件建议和提案全部办结。推动法治政府建设，实施法律顾问制度，重大决策合法性审查率100%。转变政府职能，成立市场监督管理局，完成事业单位改革分类工作。优化发展环境，分批取消、下放行政审批项目135项，非行政许可审批事项全部清零。建立权力清单和责任清单，制定完善行政权力运行流程图。推进落实“四证合一”和“一照一码”制度。成立企业投诉中心，开通网上政务服务中心。完善权力运行监督机制，发挥行政监察、审计职能，政府系统党风廉政建设取得新进展。

各位代表，2015年，我们坚持谋全局、抓关键，经济发展和民生改善同步推进，“十件大事”实现突破，“二十件实事”惠及群众。

回首极不平凡的“十二五”，我们用智慧和担当、实干和拼搏，谱写了黑河发展的新篇章。这五年，是黑河经济社会发展更加协调、发展后劲明显增强的时期，是城乡建设投入最大、面貌变化最快的时期，是民生改善最突出、群众得实惠最多的时期，经济社会发展取得显著成就。全市生产总值年均增长10%，固定资产投资年均增长17%，社会消费品零售总额年均增长14%，公共财政收入年均增长14%。五年攻坚克难、砥砺奋进，成绩来之不易，启示弥足珍贵。

第一，必须秉承符合实际的发展理念。五年来，我们始终牢牢把握黑河发展的阶段性特征，坚持跳出黑河看黑河，立足全国全省大局谋划发展，纳入“五大规划”赢得发展先机。提出坚持生态发展、实现绿色崛起的战略目标，发展“五大产业”、培育“新四型经济”的主攻方向，打造“一都三地、四位一体”魅力黑河的总体构想，坚持强基础、利长远、求创新、促发展的工作方针，推动创新型、差异化、福祉性、积累式发展的实现路径。这些理念和思路符合党的十八届五中全会精神，符合黑河发展实际，黑河始终以高于全省平均速度加快发展。实践证明，理念是行动的先导，方向比速度更重要，只要理念超前、方向正确，就能为跨越赶超精准导航。

第二，必须弘扬攻坚克难的担当精神。五年来，我们始终坚持以“攻坚战”和“工程”方式推进工作，力克瓶颈制约，实现重点突破。戮力同心战胜特大洪水，黑河大地筑起永不磨灭的精神丰碑。自我加压、敢打硬仗，超强度完成重大水利工程建设任务，公路“三年决战”里程创全省之最，“三城同创”捷报频传。多铜矿业、翠宏山铁多金属矿、中兴牧业、恒大粮油等一批大项目撑起产业骨架。实践证明，发展与矛盾总是相伴相生，只要迎难而上、敢于担当，困难和挑战就是成功道路的基石。

第三，必须打造合作共赢的开放格局。五年来，我们始终坚持有胸怀、重诚信、讲共赢，以人文交流为引领，深化对俄全方位交流合作，两地关系步入历史最好时期。以投资合作为核心，延伸跨境产业链条，夯实两地未来发展根基。以“两国一城”为依托，密切友好往来，促进情感融合，两地民众的心贴得更近。实践证明，对外开放是繁荣发展的必由之路，只有树立国际视野，打造全方位开放格局，才能赢得未来竞争优势。

第四，必须恪守朴素真挚的为民情怀。五年来，我们始终突出民生导向，把改善民生贯穿各项工作始终，75件实事让群众享有更多获得感。棚户区、泥草房和旧小区改造让居民住得更舒心，蔬菜储备库和“菜篮子工程”让群众吃上平价菜、新鲜菜，自来水复线工程保证市民饮水安全，社保扩面提标兜起民生底线，“阳光早餐”温暖环卫工人，“小绿”出行成为市民时尚……实践证明，改善民生是发展之根本，只有注重经济发展与民生改善的有机统一，才能相互促进、相得益彰。

第五，必须顺应职能转变的时代要求。五年来，我们始终把握时代脉搏，积极大胆探索，努力破除体制机制障碍，加快建设法治政府和服务型政府。下好简政放权“先手棋”，营造创新创业宽松环境，权力“瘦身”激发市场活力。打造便民服务超市，一站式服务打破审批效率低下的坚冰。厘清政府权力边界，面向社会公开“权责清单”，让权力在阳光下运行。实践证明，改革创新是发展的不竭动力，只有深化行政体制改革，发挥市场在资源配置中的决定性作用，才能释放更多发展潜能。

各位代表！五年来我们取得的成绩，是省委、省政府和市委正确领导的结果，是市人大、市政协监督支持的结果，更是全市人民团结拼搏的结果。在此，我代表市人民政府，向辛勤工作在各条战线的全市人民，向人大代表、政协委员，向各民主党派、人民团体，向驻黑部队、武警官兵和公安干警，向离退休老领导、老同志，向所有关心支持黑河发展的社会各界人士，表示衷心感谢并致以崇高敬意！

二、“十三五”时期美好愿景

“十三五”是全面建成小康社会的决胜时期。我们要深刻认识黑河发展的基本特征，增强战略定力，坚定发展信心，在全国全省发展大局中找位置、求突破，确保如期实现第一个百年奋斗目标。

当前，黑河发展仍然面临诸多问题，不可忽视，必须警醒。一是国内经济下行压力和俄罗斯经济波动，将对黑河发展产生深刻影响。二是经济扩量与结构优化、动能转换任务艰巨。三是财政拮据，难以支撑高水平民生诉求，民生改善压力很大，财源建设有待加强。四是改革进入关键期，法治政府建设任务繁重，干部队伍思想解放程度不够，不敢为、不想为、不会为现象仍然存在，人才外流严重，支撑未来发展的人才问题凸显。这些新挑战和老问题躲不开、绕不过，需要我们认真面对、全力破解。

抢抓新机遇，推动新发展，要坚定信念和决心，认清我们拥有的“五大发展优势”。一是政策优势。“五大规划”政策效应持续释放，“一带一路”、“龙江丝路带”战略带来更大红利。二是对俄优势。政府间沟通协作机制更加稳固，人文交流更加紧密，重点合作项目加快实施，特别是通道建设提速，独特的地缘优势将进一步凸显。三是生态优势。天蓝水清的生态环境，品质优良的生态产品，是生态产业发展的先决条件，生态旅游、健康养老、绿色食品将成为引领经济发展的新业态。四是资源优势。原生态的农林资源，世界级的旅游资源，富集的矿产资源，为黑河未来发展提供了强大支撑。五是农业基础优势。粮食产量实现“八连增”，农业机械化水平、土地规模经营、水利设施保障能力全面提升，为发展粮食及食品、畜产品深加工奠定了坚实基础。

今后五年，我们要高举中国特色社会主义伟大旗帜，全面贯彻党的十八大和十八届三中、四中、五中全会精神，以马克思列宁主义、毛泽东思想、邓小平理论、“三个代表”重要思想和科学发展观为指导，深入贯彻习近平总书记系列重要讲话精神，按照“五位一体”总体布局和“四个全面”战略布局，牢固树立创新、协调、绿色、开放、共享发展理念，抓住省实施“五大规划”发展战略、推进“龙江丝路带”建设契机，落实市委五届九次全会部署，坚持生态发展、绿色崛起的战略目标不动摇，坚持强基础、利长远、求创新、促发展的工作方针不动摇，坚持发展“五大产业”、“新四型经济”的主攻方向不动摇，着力优化结构，着力转换动能，着力深化改革，着力扩大开放，着力改善民生，全力打造“一都三地、四位一体”魅力新黑河、幸福新家园，力争提前实现地区生产总值和城乡居民人均收入比2010年翻一番，确保同全省、全国一道如期全面建成小康社会。

全面建成小康社会的主要预期目标是：全市生产总值年均增长7%。全社会固定资产投资年均增长8%。社会消费品零售总额年均增长7.5%。公共财政收入年均增长7%。城镇、农村常住居民人均可支配收入年均分别增长7%和8%。现行标准下农村贫困人口提前脱贫。

实现全面建成小康社会奋斗目标，需要我们以新思维、新路径引领新发展，实现新跨越。

——构建生态产业新体系。挖掘生态资源宝贵价值，推进生态工业、生态农业、生态林业、生态旅游发展，做大做强农产品深加工、高品质畜牧业、绿色食品产业，大力发展绿色低碳循环经济，培育生态产业发展新模式，用生态优势助推经济发展。

——打造创新发展新引擎。打破传统发展路径依赖，实施“互联网+”战略，推进理念、制度、管理和科技创新，实现发展动能转换。推进供给侧结构性改革，将发展方向锁定新兴领域，发展现代服务业，打造新的经济增长点。

——开创对俄合作新局面。发挥对俄区位优势和合作基础优势，加快推进跨境基础设施互联互通，构建以装备制造、能源、农林、矿产为重点的跨境产业链，以境内外园区为载体的产业聚集带，集中打造中俄能源资源合作区域物流集散中心、中俄跨境产业试验区、跨境旅游合作区和中俄文化艺术交流示范区。

——建设美丽宜居新家园。尊重、顺应城市发展规律，树立“精明增长”、“紧凑城市”理念，积极推进海绵城市、低碳城市建设，增强城市可持续发展能力。延续历史文脉、地域环境、建筑风格等“基因”，把好山好水好风光融入城乡建设，打造整洁干净宜居乡村，让黑河成为令人向往的地方。

——创造和美幸福新生活。时刻关注群众的新期盼新要求，坚持每年办好一批民生实事，让群众得到看得见、摸得着的实惠。多谋民生之利，多解民生之忧，深入推进教育领域改革、公立医院改革，加快保障性住房、福利机构建设等民生工程，打好扶贫攻坚战，不断刷新“民生高地”的惠民高度。

——树立公正为民新形象。运用法治思维和法治方式引领改革发展，推动民生改善和社会公正，深化行政体制改革，加快转变政府职能，持续推进简政放权，顺应民之所盼，强化为企服务，改善发展环境，建设勤政有为政府。

各位代表，黑河已进入蓄势崛起的新阶段。我们必须紧随时代步伐，牢牢把握发展的主动权，在转型升级上取得新突破，在发展动力上实现新转换，在补齐短板上取得新进展，不断开拓各项事业发展新局面！

三、2016年目标任务

今年是“十三五”开局之年，做好今年工作意义重大。我们要坚持稳中求进总基调，主动融入国家省发展战略，着力实施“九大工程”，为全面建成小康黑河奠定坚实基础。主要预期目标是：全市生产总值增长7%左右。全社会固定资产投资增长8%。社会消费品零售总额增长8%。公共财政收入增长6%。城镇和农村常住居民人均可支配收入均增长8%。

(一)实施“互联网+”战略工程。顺应互联网经济时代变革潮流，推动互联网与实体经济、传统产业融合发展。搭建“互联网+”平台。高度重视支持“互联网+”发展，聘请国内权威专业机构，编制电子商务发展规划。出台促进电子商务发展政策措施，引进和培育电商企业，支持电子商务服务平台建设。鼓励金融机构开展互联网支付服务，面向全国拓展网上支付、移动支付业务。加大物流企业培育力度，支持物流园区、分拨中心、仓储中心及城市末端配送快递网点建设，完善物流配送服务体系。推进电信基础设施建设。以北安新中新公司云服务平台为基础，建设大数据公共信息综合服务平台，发展服务外包业，提升电子商务信息服务水平。推动重点产业与互联网深度融合。推进“互联网+对俄贸易”，深化与阿里巴巴、京东、苏宁合作，加快黑河跨境电子商务产业园区建设，完善通关、物流、支付、结汇、退税等配套服务，力争入区电商企业数量翻一番，纳入省级电子商务示范基地。全力打造淘宝特色中国?黑河馆，年销售额翻两番。全市电商企业发展到650家以上，年销售额增长20%。支持中机、丰泰、银河等跨境电商企业发展，把中机公司打造成全省最具影响力的对俄跨境电商企业。推进“互联网+农业”，利用物联网技术，推广生态农业、智慧农业，实现种植养殖环境、检测数据与产品销售“点对点”对接，农产品质量全过程追溯，爱辉区、北安、嫩江各建1个“互联网+农业”综合服务大厅，五大连池、孙吴、逊克各建1个物联网应用示范基地，每个县(市、区)各建设5000亩以上“互联网+农业”高标准示范基地10个，其中1万亩以上4个，推动农产品由“种得好”向“销得好”转变。加快阿里巴巴集团农村淘宝黑龙江省北部运营总部网点建设，实现北安乡镇全覆盖。发挥嫩江全国“电子商务进农村”示范县引领作用，培育一批电子商务特色村和农村电商示范企业。发展智慧旅游，做好我省“旅游+互联网”黑河试点工作，建设旅游大数据分析系统、智能Wi-Fi服务及智能导服系统，实现游客中心智能电子支付全覆盖。积极探索其他领域与互联网融合发展，加速提升创新能力。

(二)实施产业项目引领工程。深入研究产业政策，紧跟市场需求，持续加大项目建设力度。安排市级重点产业项目74个，年度计划投资110亿元。狠抓项目投产达产。严格落实包保责任，全力以赴抓开工、抢进度、促投产，确保省市重点产业项目开复工率和投资完成率均达到90%以上。推进北安中粮燃料乙醇、五大连池科迪矿泉水等41个项目开工建设，力争北安象屿金谷玉米深加工、逊克东安岩金矿等33个项目建成投产，尽快形成产业支撑。加强项目谋划，根据市场需求和区域优势，与大专院校、科研院所深入对接，提升项目谋划针对性和专业水准。创新融资方式。充分发挥产业引导股权投资基金作用，鼓励中小企业参与资本市场运作，建立企业上市孵化基地，推进五大连池名镇、金禾经贸、中兴牧业等企业上市工作，力求上市融资取得新突破。做好PPP项目储备工作，通过PPP机制吸引民间资本投入。推进我市农村物权融资平台及民间借贷登记中心试点工作，继续推进农村信用信息体系建设。强化政银企对接，引导金融机构创新产品，支持地方实体经济发展，金融机构为企业投放贷款增长10%以上。向上争取到位资金增长10%以上。提升园区承载能力。突出优势主导产业，合理规划项目布局，引导同源、同类企业和关联度较高的项目加快向园区集聚，打造创新型特色产业集群。积极争取政策资金支持，加大投入力度，高标准完善园区功能，提升黑河进出口加工产业园、黑河(北安、孙吴)健康产业园、嫩江工业示范基地、五大连池矿泉工业园区承载能力，搭建产业集聚平台，带动区域经济加快发展。增强招商引资实效。坚持“九个更加注重”，引资与引智并举，围绕产业链延伸和科技成果转化，引进战略投资者，实施一批大项目、好项目。创新招商方式，全面梳理招商线索，实行园区招商、以商招商、企业招商、小分队招商等多种有效形式，主动出击跟踪招商，开展“招商之冬”等活动，巩固扩大招商成果。提高签约项目落地率和资金到位率，到位资金增长20%。支持民营经济发展，培育做大民营经济。

(三)实施现代农业提质工程。创新生产经营模式，提高农业现代化水平，促进农业增效和农民增收。优化种植结构。坚持稳豆、增麦、调控玉米、大力发展经济作物。玉米调控在550万亩左右，开发青贮玉米、专用玉米生产。恢复小麦和燕麦在100万亩左右，加快发展优质特色强筋型春小麦。稳定绿色有机大豆、芸豆、土豆生产，大豆稳定在1000万亩左右，引进培育黑豆、小粒豆等专用品种。发展蔬菜、饲草、北药和浆果等特色经济作物，种植面积达到90万亩。扩大绿色、有机、无公害农产品种植和推广，绿色有机种植面积310万亩。大力发展畜牧业。实施畜牧业发展三年规划，突出发展“两牛一猪一羊”，扩大养殖基地规模，新建标准化牧场11个。根据区域特点，积极发展鹿、马、鹅、野猪、矿泉鸭等特色养殖。加强重大动物疫病防控，保障畜产品质量安全。发展标准化水产品养殖，渔业产值增长8%。提高经营水平。发挥加工企业龙头作用，加快中兴牧业万头奶牛养殖场和乳制品深加工项目建设，奶牛存栏3万头以上，日产鲜奶300吨。扶持北安宜品乳业、嫩江辰鹰乳业和鸿兴面粉、五大连池开发面业等企业发展，推进爱辉区北丰进口大豆加工、孙吴恒大粮油等项目建设。完善农产品产业链条，提高转化率和增值率，扩大绿色食品产业占工业增加值比重。打造特色品牌。突出寒地黑土、绿色有机、原生态，支持中兴、瑷珲等已有品牌发展壮大，加强品牌整合，集中培育打造北纬49度生态农业、矿泉产品和硬红春小麦、非转基因大豆、大小兴安岭山珍等高纬寒地特色品牌。强化基础保障。完成省亿亩生态高标准农田建设任务，有序推进土地整治，提升优质耕地比重，提高粮食生产能力。推进水利设施现代化进程，实施7类34项重大水利工程，推进水电站增效扩容和水库除险加固工程建设，实施旱田节水灌溉和灌涝区治理。提高现代农机合作社建设和经营水平，新建现代农机合作社6个，创建市级规范社10个、省级规范社6个。

(四)实施对俄合作升级工程。坚持以构建互联互通网络为基础，以投资合作为核心，以人文交流为引领，推动对俄合作向全方位、高层次、宽领域转型升级。建设开放通道。加强协调争取，力促黑龙江大桥、跨江空中索道项目开工。五大连池机场主体工程完工，争取试飞。推动北黑铁路改造升级和北五铁路建设工程有实质性进展。积极向上申报，开通黑河国际空港。推进嫩江通用机场项目建设。打造合作平台。编制桥头区、跨境园区规划，为建设国家级跨境经济合作区奠定基础。做好黑河保税物流园区(B型)申报工作。加快境外工业园区、石化建材加工园区、中俄农业产业园区等园区建设，打造跨境产业链，构建境内外园区协调呼应、产业互补的发展格局。优化贸易结构。扩大汽车、工程机械、地工产品出口，电力、农产品进口规模。争取游客进出境随身携带包裹和民贸进口商品纳入海关统计。增加互市贸易交易点进口商品种类，扩大销售规模，努力将我市打造成民贸商品集散地和销售中心。加强投资合作。继续实施“走出去”战略，支持国内企业参与俄资源能源合作开发。阿穆尔—黑河边境油品储运与炼化综合体项目开工。继续推进萨哈油田区块和云津、首钢在俄铁矿开发等重大合作项目，力争取得实质进展。深化人文交流。加强与阿州及布市交流互访，增进了解互信。深化教育、科技、医疗、体育等领域交流，举办2016中俄双子城展览会，扩展合作领域。密切友城交往，加强与俄腹地城市沟通联系，增加友城数量。深入研究俄政策法规，为企业提供信息咨询服务。

(五)实施文化旅游精品工程。坚持用文化提升旅游内涵，以旅游带动文化产业发展，打造新型消费热点。推动文化产业发展。编制文化产业发展总体规划。办好第七届中俄文化大集，扩大品牌影响力。推动中俄风情文化旅游驻场演出提档升级，打造国内文化旅游地标性文化产品。改扩建旅俄华侨纪念馆，重新布置展陈，传承历史记忆。加快推进中俄民族风情园、中俄文化创意产业园、中俄画家村、冰雪艺术馆、北方影视城、黑河生态馆等文化载体建设，将黑河打造成俄油画集散地、中国冰雪画创作基地。积极发展影视业，力争引进1—2部影视剧在黑河拍摄。加强非物质文化遗产保护，传承地域文化精华。弘扬珠宝玉石文化，推进黑河珠宝玉石加工区项目，建设宝玉石加工厂和保税库。巩固逊克玛瑙在北红玛瑙中的主体地位，放大名片效应，培植龙头企业，建成逊克玛瑙文化产业园。提升旅游品牌形象。创新旅游营销模式，继续通过机场、高铁、龙广之声等加大线上线下旅游营销力度。积极推进黑河—布市跨境旅游合作区试点申报工作。利用黑河旅游中俄文数字化平台和阿州旅游网，发布两地旅游信息，实现游客互送。设计旅游产品组合，打造自驾、自助旅游精品线路。拓展境外旅游线路，争取开通赴俄腹地城市航线及韩国旅游包机。建设瑷珲—腾冲文化旅游合作发展联盟，筹建人口地理公园，打造“瑷珲—腾冲线”文化旅游长廊。开展“精品景区提升年”活动，集中打造五大连池精品景区，加大开发保护力度，推进格拉球山、卧虎山、火山博物馆等项目建设，完善新镇设施，改造老镇环境，积极探索低空旅游飞行项目。加大旅游产品供给，形成新的经济增长点和核心竞争力。争取孙吴侵华日军罪证陈列馆、嫩江墨尔根古道驿站博物馆晋升3A级景区。发展健康养老产业。推进中俄国际养生城、五道豁洛康疗养生岛、五大连池养老城等项目，鼓励养老机构与上海、浙江、三亚等地养老机构合作，打造五大连池“候鸟式”矿泉康疗养老、黑河—五大连池—布市跨境旅居养老模式，推进医养结合型养老机构发展。积极开展异地旅居养老网络推介、机构养老智能管理、社区居家养老智能服务，在市第二福利院试点建设养老智能管理服务平台系统。

(六)实施城市品位提升工程。统筹推进城市规划、建设和管理，巩固国家卫生城、国家园林城创建成果，做好国家环保模范城验收和全国文明城创建工作，不断提高城市发展系统性、持续性和宜居性。坚持规划引领。高起点、高标准编制市区控详规划。开展城市近期建设规划编制和重点地块城市设计，编制海绵城市、城市综合管廊、口岸建设布局规划。加强规划实施监督，确保工程建设按规划实施。完善城市功能。继续推进市区道路、广场等项目建设，黑河沿江公园改造完工，新建“黑河眼”观光塔，完成双子城广场建设，实施大黑河岛植被还绿工程。加快棚户区改造，加大城中村、烧火楼、泥草房改造力度。继续推进“三供三治”项目建设，对部分街路老旧供热、供水管网进行改造。进一步提升城区绿化美化净化能力，保持城区常绿常新。加快完善各县(市)城市功能，改造完成北安庆华主题公园，新建嫩江城东垃圾处理场，实施逊克滨江路改扩建工程。加强信息化技术应用，完善智慧城管设计，加快硬件和平台建设，打造智慧城市。认真贯彻《中共中央国务院关于深入推进城市执法体制改革改进城市管理工作的指导意见》，进一步提高城市管理和公共服务水平。加快新型城镇化和美丽宜居乡村建设。坚持新型城镇化与农业现代化同步发展，发育特色主导产业，完善配套基础设施，建设富有活力、各具特色的新型城镇，打造生态宜居、幸福和谐的美丽乡村。合理引导农业人口有序向城镇转移，稳步推进城镇基本公共服务常住人口全覆盖，提高户籍人口城镇化率。增强示范带动效应，加快15个城镇化试点镇建设，重点提升改造44个村，打造中俄界江风情、历史文化民俗展示、城乡一体化、合作共建、火山景观、生态田园6条美丽宜居乡村示范带。

(七)实施“黑河蓝”保护工程。倍加珍惜良好的生态环境，加大生态保护和环境治理力度，让黑河的蓝天白云常在，绿水青山永驻。深入推进“一退三还”。争取上级资金和政策支持，搞好规划设计和地块落实，还林、还草、还湿面积15万亩。结合“一退三还”，推进嫩江、孙吴、逊克等北药生产基地建设，加大野生蓝莓保护和寒地小浆果繁育力度，新增北药种植面积5万亩、蓝靛果忍冬等寒地小浆果繁育面积1.2万亩。扩大嫩江西伯利亚杏生态经济试验林规模，促进沙化林地植被恢复。加大造林护林力度。实施新一轮造林绿化行动，完成造林20万亩、森林抚育40万亩。加快北部重点火险区综合治理及黑河、嫩江航空护林站综合改造工程建设，健全森林防火网格化指挥体系，力争不发生重特大森林火灾。推进黑河森林生态系统定位观测研究站建设。强化生态环境治理。加强重点行业环境监管，提高节能减排能力。推进孙吴海峰热电脱硝项目建设，做好五大连池风景区、五大连池龙镇污水处理工程环保验收工作。实施“三减”行动，在农药、化肥、除草剂亩施用量比省平均低20%、14%、30%基础上，提前实现“零增长”目标。扩大测土配方施肥和作物秸秆还田面积，减轻农业面源污染。落实最严格的水资源管理制度，推进水土保持项目建设，开展重点河流违规采砂和清障专项治理。

(八)实施改革创新攻坚工程。树立改革思维，提高创新能力，推动经济社会发展。深化重点领域改革。贯彻“两大平原”现代农业综合配套改革试验工作部署，扩大农村土地确权登记试点范围。引导新型经营主体规范发展，各类经营主体和各自规模经营面积分别增长10%以上。推进国有林场改革，稳妥解决历史遗留问题，实现生态建设、产业发展和职工生活改善共赢。推进办学管理体制改革，推行爱辉区教育集团化、学区制、联盟发展模式。探索教师编制改革，试点“县管校用”义务教育学校教师管理制度，统一配置师资，实行无校籍管理。稳步推进医药卫生体制改革，巩固提高县级公立医院改革成果。落实全面两孩政策，改革完善计划生育服务管理。推进行政体制改革。加大行政审批改革力度，科学编制行政审批流程和审批事项办事指南，对国家、省、市取消下放的行政审批权力，涉及几个部门关联审批的，做到权力同级下放、同步合拍。加强下放权力承接能力建设，确保接得住、管得好。建立行政审批责任监督机制，强化事中事后监管。继续推进“两集中、两到位”改革，实现行政审批部门应进必进、充分授权，所有非税收费集中统一收缴。完善网上政务服务中心建设，逐步实现所有政务服务事项网上集中审批和管理。整合工程招投标、土地使用权和矿业权出让、国有产权、政府采购等交易市场，建立公共资源交易监管平台，加快推进交易全过程电子化，实现交易全流程公开透明。推进行政复议体制改革，全面实行行政复议权相对集中。全力优化发展环境。创新政府管理理念、管理机制，提升为企服务能力。推广北安经验，实行“审批零障碍、服务零距离”模式，优化“一个窗口受理、一站式审批、一条龙服务”审批流程，建立“三减两联合”制度，推动项目审批提速。优化口岸联检部门工作流程，提高通关效率。清理规范中介机构，最大限度减少对企业干预。加强行政执法监督，落实行政执法责任制。规范市场环境秩序，开展专项整治行动。发挥企业投诉中心作用，拓宽投诉渠道，严厉查处破坏和影响发展环境案件。

(九)实施民生改善幸福工程。守住底线、突出重点、完善制度、引导舆论，加大财政资金投入，让群众过上更好生活。突出抓好就业创业工作。坚持就业优先战略，推进大众创业、万众创新，实施更加积极的就业政策，加大对灵活就业、新就业形态支持力度，进一步加强创新创业服务平台建设。全市新增城镇就业1.3万人，城镇登记失业率保持在4.5%以内。深入实施“促进30周岁以下高校毕业生就业工程”，提升高校毕业生就业率。加快发展各项社会事业。启动对俄科技合作服务平台建设，搭建资源共享载体。统筹各类教育协调发展，提高办学质量效益，缩小区域、城乡和校际间办学差距。全市二级以上医疗机构实行医疗责任险，完善疾病应急救助制度。提升农村及边远地区医疗服务能力。发挥中医药特色优势，与布市、雅库茨克市医疗机构建立友好医院关系。加大农村乡镇广电网络改造力度，力争有线电视网络全覆盖。提升社会保障能力。全面实施机关事业单位养老保险制度和城镇居民大病医疗保险制度，加快推进城镇职工(居民)基本医疗保险市级统筹，进一步落实失业保险支持企业稳定岗位政策，全面实施工伤保险基准费率和浮动费率办法。完善重特大疾病医疗救助制度，医疗救助比例提高到75%。做好农村救济性扶贫工作，对丧失劳动能力贫困人员实施兜底保障。维护社会安全稳定。完善安全生产责任体系，建立综合监管信息平台。进一步加强食品药品安全监管。推进信访制度改革，拓宽信访反映渠道，开展网上信访工作，解决群众合理诉求。加强应急演练，提高应对突发事件、重特大自然灾害处置能力。完善社区矫正数字化管理系统。深入推进“平安黑河”建设，保持社会治安和公共安全形势稳定。凝聚军民融合发展深度共识，强化融合发展制度保障。做好新形势下双拥工作。加强国防后备力量建设。推进边境基础设施信息化建设，实现边境监控全覆盖。加强反恐力量建设，强化应急处突能力，维护边境秩序稳定。同时，进一步做好老区、民族宗教、侨务、妇女儿童、人防、档案、地方史志等工作。

各位代表，实现全面建成小康社会奋斗目标，既要谋大事以致远，又要行实事以利民。今年，我们要持之以恒，继续抓好事关长远、牵动全局的“十件大事”，全力办好惠及民生、增进福祉的“二十件实事”。

——十件大事。(1)力促黑龙江大桥、跨江空中索道项目开工。(2)五大连池机场主体工程完工，争取试飞。推进黑河国际空港开通。(3)三江治理和黑河四岛保护开发工程全面竣工。(4)北黑铁路改造升级和北五铁路建设工程有实质性进展。(5)做好黑河保税物流园区(B型)申报工作。(6)黑河沿江公园改造完工，新建“黑河眼”观光塔，完成双子城广场建设。(7)启动大黑河岛东侧桥梁和悬索桥工程。(8)编制海绵城市和城市综合管廊建设规划。(9)巩固国家卫生城、国家园林城创建成果，做好国家环保模范城验收和全国文明城创建工作。(10)实施精准扶贫精准脱贫，推进26个村整村扶贫攻坚工程，减少贫困人口3.3万人。

——二十件实事。(1)改善居民住房条件，市区完成15年以上老旧住宅楼房维修，改造清真寺周边平房及7栋烧火楼。发放公共租赁住房补贴4800户。全市改造农村泥草(危)房4640户。(2)改善群众生活环境，市区改造供水管网5.2公里、老旧排水管线1公里、老旧供热管网15.7公里，新建移动式生活垃圾压缩站3座。(3)改善群众生活条件，在具备条件的新建小区开展燃气入户试点工作。(4)打造整洁有序城市环境，归集整理50个小区通讯管线。(5)提高义务教育均衡发展水平，创建义务教育标准化学校7所，孙吴、逊克完成国家义务教育基本均衡发展县建设任务。(6)推进教育“三通两平台”建设，利用数字教育资源开展教学活动教师达到70%以上，建立个人网络学习空间的学生和教师比例分别达到40%、60%以上，推进“专递课堂”、“名师课堂”、“名校课堂”建设。(7)在全市学校开展光生物教学护眼灯试点工作，降低学生视力不良率。(8)创建平安和谐校园，开展校园周边环境专项治理。(9)改善医疗服务环境，市第一人民医院改扩建、儿科楼主体工程和中医医院附属续建工程完工。(10)提升卫生保障水平，新型农村合作医疗人均筹资标准由470元提高到560元。(11)提高社会救助标准，建立低保标准自然增长机制，城乡低保标准提高10%以上。(12)加快体育设施建设，市全民健身中心体育馆主体工程完工，黑河体育公园投入使用，新建3个多功能运动场。(13)加强社会福利机构建设，开工建设市精神病人福利院，全市养老服务床位数达到6800张，每千名老年人拥有床位数达到31张。(14)提高社区建设水平，创建城市示范社区5个、农村示范社区5个，争创国家级减灾示范社区3个、省级减灾示范社区4个。新建农村社区幸福院56家。(15)提升餐饮服务和食品安全保障水平，全市完成100家餐饮服务单位“明厨亮灶”改造。(16)推进饮水安全工程，新建农村饮水安全工程50处，解决3万人饮水不安全问题。(17)在全社会普及防火、防震知识，开展群众性应急避险演练，依托学校和体育场馆等设施，建设室内应急避难场所。(18)方便两地边民绿色出行，协调俄布市实现公共自行车“一卡通”。(19)缓解群众出行压力，在市区增设立体、绿荫停车场。(20)提升便民服务承载能力，为民服务大厅投入使用。

各位代表，做好今年工作，必须加强政府自身建设。我们要坚决贯彻落实市委决策部署，自觉接受人大依法监督和政协民主监督，认真听取各民主党派、工商联以及无党派人士意见建议。加强法治建设，落实“谁执法、谁普法”责任制。坚持科学民主决策，严格按法定程序、法定权限行使职权，建设法治政府。厉行勤俭节约，进一步压缩一般性支出和“三公”经费，财政支出更多向民生倾斜。巩固“三严三实”专题教育成果，深入治理庸政懒政怠政行为，加大对重点领域、重点项目、重大资金审计监督力度，加强政府系统党风廉政建设。各位代表，蓝图已经绘就，征程任重道远，时代赋予黑河跨越发展的历史机遇，也赋予我们光荣艰巨的神圣使命。让我们在省委、省政府和市委的坚强领导下，紧紧依靠全市人民，始终保持改革创新、奋发有为的精神状态，始终保持重任在肩、不辱使命的责任担当，为全面建成小康社会而努力奋斗！
